# Situations Leading to Anterior Cruciate Ligament Injury in Sports: A Systematic Review with Meta-analysis

**DOI:** 10.1007/s40279-026-02436-1

**Published:** 2026-04-18

**Authors:** Patrick Mai, Steffen Willwacher, Lina Rahlf, Tim Hoenig, Luca Braun, Carlo von Diecken, Kevin Bill, Dominik Fohrmann, Tron Krosshaug, Karsten Hollander, Thomas Gronwald, Jan Wilke

**Affiliations:** 1 Institute for Advanced Biomechanics and Motion Studies, Offenburg University, Badstr. 24, 77652 Offenburg, Germany; 2 Department of Physical Performance, Norwegian School of Sport Sciences, Oslo, Norway; 3 Institute of Sports Science, Europa-Universität Flensburg, Flensburg, Germany; 4 Department of Trauma and Orthopaedic Surgery, University Medical Center Hamburg-Eppendorf, Hamburg, Germany; 5 Insititute of Biomechanics and Orthopaedics, German Sport University Cologne, Cologne, Germany; 6 Institute of Interdisciplinary Exercise Science and Sports Medicine, MSH Medical School Hamburg, Hamburg, Germany; 7 Department of Sports Medicine, Norwegian School of Sport Sciences, Oslo Sports Trauma and Research Center, Oslo, Norway; 8 Center of Sports Science, University of Bayreuth, Bayreuth, Germany

## Abstract

**Background:**

Anterior cruciate ligament (ACL) injuries are common in many sports and impose substantial performance and long-term health burdens. A quantitative synthesis of real-world, video-identified game situations can inform sport-specific prevention methods.

**Objective:**

The aim of the study was to identify game situations and movement patterns leading to ACL injury in different sports.

**Design:**

Systematic review with meta-analysis.

**Data Sources:**

PubMed, Google Scholar, Web of Science, and Scopus were searched for studies analyzing video recordings of ACL injuries sustained by athletes of any sex during training or competition. Random effects meta-analyses of prevalence and moderator analyses (sport as a factor) were performed.

**Results:**

In total, 39 articles (1551 video-verified ACL injuries) of on-average moderate quality (Quality Appraisal for Sports Injury Video Analysis Studies [QA-SIVAS]) scale mean 66%) were included. Noncontact (*n* = 745), indirect contact (*n* = 533), and direct contact (*n* = 273) mechanisms were reported, with notable sport-specific differences. Across all injuries, the most frequent contexts were ball action (45.7%), pressing/tackling (40.9%), and cutting (36.6%). Within noncontact cases, cutting (53.8%), pressing/tackling (50.2%), decelerating (38.9%), and landing (30.1%) were the most prevalent actions, whereas being tackled was most frequent in indirect contact cases (56.1%). For direct contact injuries, pooled action-specific estimates were available for being tackled (23.9%) and pressing/tackling (24.2%). Injuries typically occurred at high horizontal speed (53.8%; noncontact 70.7%), were more frequent during ball possession (67.5%) and offensive play (55.4%), and happened more early in time within the first 25% of the game in football (37.5%) and netball (37.8%).

**Conclusions:**

The identified patterns support the use of mechanism-specific, sport-tailored prevention strategies (e.g., technique/strength, neurocognitive functioning for noncontact, perturbation-based drills for indirect contact, and rules/choice of equipment for knee-directed contact). Methodological improvements, such as harmonized and more detailed injury reporting, are needed to refine risk estimates.

**Supplementary Information:**

The online version contains supplementary material available at 10.1007/s40279-026-02436-1.

## Key Points

**Table:** 

This systematic review with meta-analysis provides a quantitative synthesis of video-verified anterior cruciate ligament (ACL) injury situations across sports (39 studies; 1551 injuries)
Across sports, the most frequently identified injury-inciting actions involved ball-related play, defensive pressing/tackling, and cutting; noncontact injuries clustered around cutting, deceleration, and landing, whereas being tackled predominated in indirect contact cases (with limited action-specific data available for direct contact injuries)
ACL injuries commonly occurred during ball possession and offensive play and were often associated with high horizontal speed, supporting mechanism-specific, sport-tailored, scenario-based prevention strategies and underscoring the need for more harmonized, injury-level reporting in future video studies

## Introduction

Anterior cruciate ligament (ACL) ruptures count among the most devastating injuries in sports. Annual injury incidence ranges from 0.15 to 3.67% in professional athletes and from 0.03 to 1.62% in amateur athletes across various sports [1]. When expressed relative to athlete exposures, female athletes exhibit a higher incidence rate of 1.5/10,000 compared with men, at 0.9/10,000 [2]. Furthermore, there is a high risk for a second ACL injury (pooled incidence: 16.9%; 8% for contralateral ACL injuries and 10% for ipsilateral ACL injuries [3]). Ruptures of the ACL are typically associated with long (around 9 ± 2 months [3]) return to sport (RTS) times or unsuccessful RTS, functional impairments, and potential long-term health consequences such as osteoarthritis [4, 5], overall leading to a significant burden and associated societal costs [6].

ACL injuries can be categorized as direct contact, indirect contact, or noncontact traumas [7]. Contact injuries result from direct blows or tackles to the proximal lower leg, knee, or distal thigh. Indirect contact injuries occur without direct force application to these regions (e.g., push on the upper body or contact to the foot). Noncontact injuries are sustained without any physical contact with another player. Differentiating between contact mechanisms is essential because preventive strategies for specific mechanisms differ substantially. While noncontact injuries can be reduced through neuromuscular training and technique modification, direct contact injuries often require sport-specific rule changes or the use of protective equipment; thus, accurate classification directly informs the design of targeted prevention measures [7, 8].

Although exercise programs have been demonstrated to be effective for ACL injury prevention, they may be further optimized by precisely identifying the specifics of the injury scenario [9]. Knowing the game situations (e.g., offensive/defensive play, ball possession, early/late game) and movement patterns (e.g., cutting, landing) performed in the moment of injury, as well as potential dependencies on the type of sport, would allow for the development of more specific prevention approaches.

Different sports are characterized by distinct movement patterns and constraints, including, e.g., variations in intensity, relative proportions of linear and nonlinear motions, technical demands, visual–spatial requirements, or the degree of physical contact. Understanding the differences and similarities between situations that lead to ACL injuries across various sports enables the identification of both universal risk factors and sport-specific mechanisms.

Therefore, identification of the most frequent injury-inciting situations has direct implications for targeted prevention strategies. For sports governing bodies, evidence of high-risk actions under specific contextual conditions may inform rule modifications or equipment standards designed to better protect athletes in critical situations. For coaches and practitioners, such knowledge helps prepare athletes to cope with high-risk scenarios by integrating specific drills and strengthening programs aimed at improving both technique and the capacity of biological structures to protect the ACL. For researchers, the synthesized situational patterns provide a framework for, e.g., detailed biomechanical analyses. Such analyses can clarify which structures are most critically loaded at the time of injury. They can evaluate the efficacy of preventive interventions, ranging from targeted training drills to innovations in footwear or protective equipment, under conditions that realistically reflect the injury mechanisms observed in a sport.

Video analysis provides direct observation of real-world injury events—including, e.g., task demands, opponent interactions, and unanticipated perturbations—that cannot be ethically or reliably reproduced in the laboratory and are prone to recall bias when obtained retrospectively from athlete interviews [10]. As a complement to laboratory biomechanics and prospective epidemiology, video analysis is now a rigorously appraised method [11] with growing adoption across sports in recent years, offering the potential to synthesize insights on injury situations through a systematic review and meta-analysis. While systematic reviews on ACL rupture situations have been conducted previously [12, 13], they did not include the large number of articles published in the last 2 years (2024 and 2025). Furthermore, one review neither systematically analyzed situational patterns nor performed a quality rating of the included studies [12]. Additionally, neither of the published systematic reviews performed a meta-analysis to synthesize the findings [12, 13].

Consequently, the purpose of this systematic review with meta-analysis was to comprehensively analyze video-identified ACL injury patterns across different sports. Our objectives were (a) to quantify the proportions of noncontact, indirect contact, and direct contact ACL injuries with respect to characteristics of game situations and specific movement patterns and (b) to identify potential differences between sports.

## Methods

A systematic review with meta-analysis adhering to the Preferred Reporting Items for Systematic Reviews and Meta-Analyses (PRISMA) guidelines [14] was performed. The review was prospectively registered in the International Prospective Register of Systematic Reviews (PROSPERO) database (CRD42022337340).

### Literature Search

Six investigators (S.W., P.M., K.B., L.B., C.V.D., and D.F.) identified relevant articles using PubMed, Google Scholar (first 200 entries), Web of Science, and Scopus. The Google Scholar search was conducted in incognito mode to avoid personalized results. The search was performed on 3 April 2023 and updated on 18 August 2025. We used the following string:

(case* OR mechanism* OR situation* OR event* OR characteristic* OR movement OR injur* OR scenario* OR rupture* OR torn OR pattern*) AND (ACL OR “anterior cruciate ligament”) AND (video-based OR “video analysis” OR 2D OR 3D OR footage OR televi* OR TV OR recording* OR tapes).

Search results were uploaded to the web interface of rayyan.ai [15]. After removing duplicates, four independent team members screened the titles and abstracts (S.W., P.M., L.B., and C.V.D.). Articles were eligible if (1) published in English or German in peer-reviewed journals, (2) used video analysis to analyze ACL injuries sustained during training or competition, and (3) reported movement patterns performed during the moment of injury. We excluded all articles meeting the following criteria: (1) randomized controlled trial design, case report, or review, (2) analysis of other injuries than ACL rupture, (3) analysis of ACL injuries during nonsporting activities, (4) investigation of nonhuman subjects, (5) in vitro or cadaver studies, (6) execution of a post-injury analysis, and (7) analysis of injury situations by methods other than video recordings.

Eligible studies were discussed regarding inclusion among the review team. Disagreements were resolved through consultation with an additional investigator (D.F.). Full texts were screened using the same inclusion and exclusion criteria. Additional sources were identified through reference lists and a co-citation method using the bibliographic coupling concept (www.connectedpapers.com).

### Data Extraction

Five investigators (T.H., D.F., K.B., P.M., and K.H.) independently extracted data from the eligible studies. This included study characteristics (design, source of video recordings, duration, and country of data collection), population details (sex, player experience, performance level, and type of sport), and the frequencies of noncontact, indirect contact, and contact ACL injuries. The data extraction was conducted overall for each study, as individual injury data were not reported in many articles. We used the definition of Luig et al. [16] to categorize the injury mechanism into noncontact, indirect contact, and direct contact.

In several of the included studies, the description of ACL injury events was limited to the executed movement, without further detail on the broader situational context. To account for this, we distinguished between movement patterns (e.g., change of direction) and situational patterns (e.g., ball possession). As a result, a single injury case could be categorized simultaneously under both a situational and a movement pattern. Consequently, overlap between categories was unavoidable, which means that aggregated proportions across categories may not sum precisely to 100%.

For the *movement pattern* during the moment of injury, we used the following categories: landing (single- and double-leg landing), cutting (i.e., spontaneous, quick change of direction/sidestepping), decelerating, accelerating, ball delivery (kicking, passing, heading), dribbling, tackling/pressing, and being tackled. Tackling/pressing refers to defensive actions to regain possession of the ball, while being tackled refers to an offensive action where the injured player is tackled by a defender. *Movement speed* was classified as none (standing), low (walking/jogging), or high (running/sprinting) [17–27]. Regarding the *game situation*, we distinguished between ball possession (yes/no) and tactical situation (offensive/defensive). In addition, the timing of injuries was examined. Injuries were categorized as occurring in the first, second, third, or fourth quarter of a match. For sports without quarter timing (e.g., football, which uses halves), match durations were converted into quarters.

Regarding the participants in the primary studies, we classified their expertise level from 1 (recreational) to 5 (world-class) according to the framework of McKay et al. [28], which utilizes training volume and performance metrics.

We did not consider biomechanical descriptions of ACL injuries in our analysis.

### Study Quality Assessment

Risk of bias and methodological quality of the video analysis studies were assessed by three independent investigators (L.R., T.H., and T.G.) using the Quality Appraisal for Sports Injury Video Analysis Studies (QA-SIVAS) scale [11]. The QA-SIVAS scale exhibits high reliability and construct validity for evaluating video analysis studies of musculoskeletal injury. The instrument consists of 18 distinct items, each scoring 0 (no/not stated) or 1 (yes/present). The maximum score is 18, and the quality rating is a percentage value (reached score/maximum score (%)).

### Data Synthesis and Statistics

Weighted summary proportions with 95% confidence intervals (CIs) were pooled using meta-analysis of prevalence for the respective movement pattern, movement speed, and game situation variables. We performed separate analyses for (1) all injuries combined, (2) noncontact, (3) indirect contact, and (4) direct contact ACL injuries. As not all included studies consistently reported data using all three subcategories, we also provide pooled data on combinations of direct and indirect, as well as indirect and noncontact injuries, as a supplement (Supplementary Online Material).

Statistical analyses followed established workflows for meta-analysis of prevalence as described by Barendregt et al. [29]. For each study and variable, the frequency of an ACL injury situation or movement pattern was expressed as a proportion (event count divided by the number of injuries with available information for that variable). To obtain summary estimates that are generalizable across studies while acknowledging that underlying “true” proportions may vary between studies owing to clinical and methodological heterogeneity (e.g., sport, playing level, video source/quality, and operational definitions of injury situations), random effects meta-analyses of prevalence were applied. Because inverse-variance pooling of raw proportions can yield unstable variance estimates when proportions approach 0 or 1, study-specific proportions were variance-stabilized using the Freeman–Tukey double arcsine transformation prior to pooling. Between-study variance (*τ*^2^) was estimated using a DerSimonian–Laird approach and incorporated into inverse-variance random effects weights (1/(*v* + *τ*^2^)), such that study weights reflected both within-study sampling variance and between-study heterogeneity. Summary prevalence estimates and corresponding 95% confidence intervals were calculated on the transformed scale and subsequently back-transformed to the proportion scale; statistical heterogeneity was quantified using Cochran’s *Q* and *I*^2^ statistics.

In addition to pooled prevalence estimates, moderator analyses were performed using sport type as a factor and proportions as the dependent variable within a random effects meta-regression framework on the transformed scale, applying the same *τ*^2^-based weighting approach [29]. *P* values < 0.05 were considered statistically significant. Moderator analyses were conducted only when at least three studies were available. For sports represented by two studies, mean proportions are reported without moderator testing, and for sports represented by a single study, the study-specific proportion is reported descriptively.

Analyses were performed using custom MATLAB scripts (MathWorks, Natick, MA, USA) and jamovi (the jamovi project (2023), jamovi, version 2.3.28).

### Equity, Diversity, and Inclusion Statement

Equity, diversity, and inclusion were strategically targeted for the present study. The research and author team consists of female and male researchers with varying nationalities, career levels, professions, and fields of expertise (e.g., exercise science, biomechanics, sports medicine, and trauma surgery). Regarding inclusion criteria for study populations, a diverse group from all geographic locations, sports, and sexes was targeted. However, the study population data included were mostly from high-income countries with no representation of low-income countries.

## Results

### Search Results

**Fig. 1:**
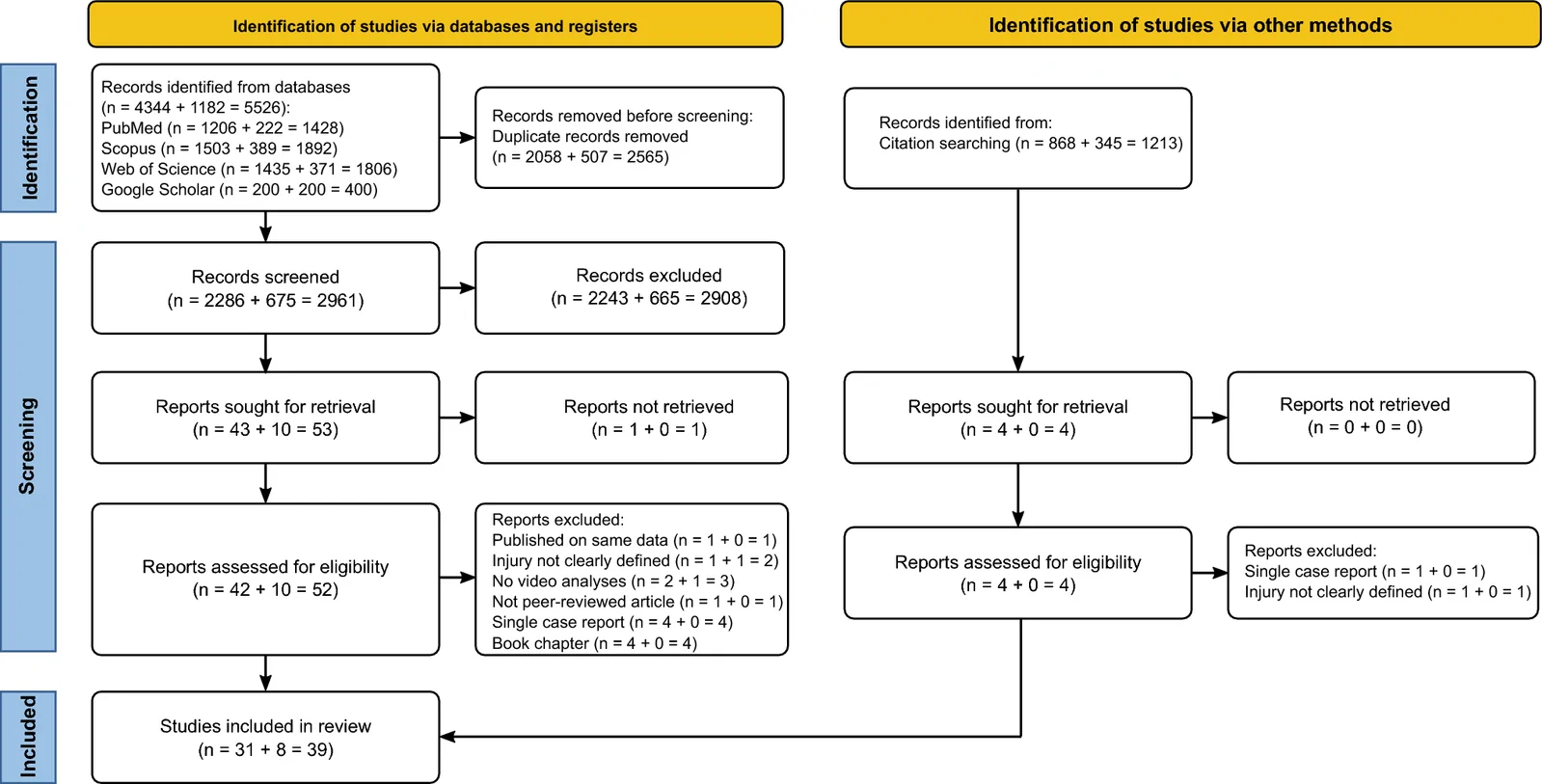
Flow diagram of the literature search and study inclusion process. Numbers in brackets indicate the number (*n*) of the initial search and the updated search, resulting in the overall number (*n* = initial search + updated search = overall number)

Database searches returned a total of 5526 articles. After removal of duplicates, 2961 were checked for inclusion. Two studies reported on the same data set [30, 31]; thus, one [31] was excluded from the meta-analysis. The final sample of eligible papers consisted of 39 articles (Fig. 1).

### Characteristics of Included Studies

**Fig. 2:**
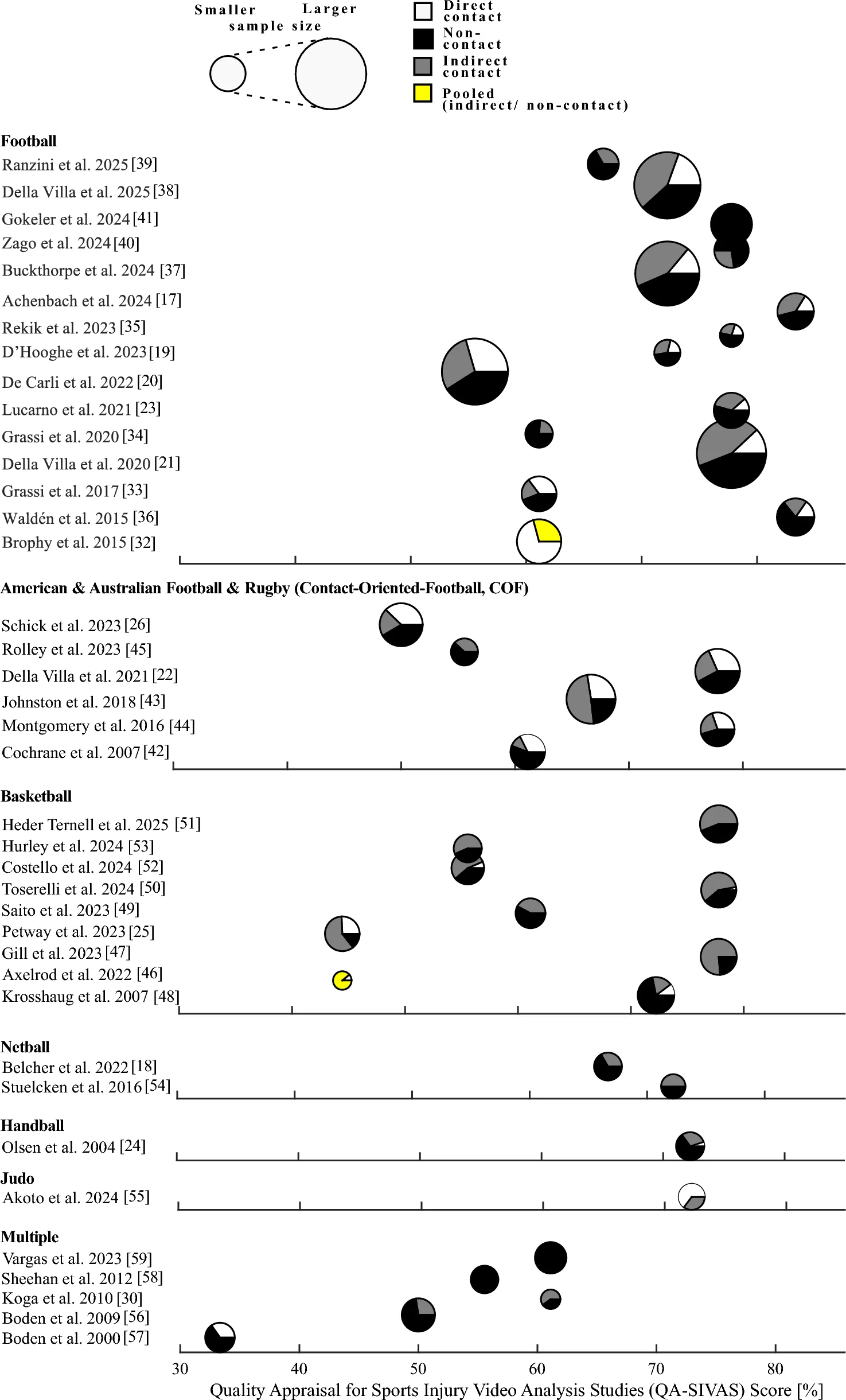
Pie charts (scaled to the number of ACL injuries) displaying the Quality Appraisal for Sports Injury Video Analysis Studies (QA-SIVAS) score of the included studies, stratified by the type of sport

Overall, 34 of the 39 studies reported ACL injuries in a single sport (Table 1; Fig. 2). Of these, 15 focused on football (soccer) [17, 19–21, 23, 32–41], 6 on American Football and Australian Rules football or rugby (summed as contact-oriented football [COF]) [22, 26, 42–45], 9 on basketball [25, 27, 46–53], 2 on netball [18, 54], and 1 each on judo [55] and handball [24] (Table 1). The remaining five articles reported ACL injuries in multiple sports (Table 1) [30, 56–59]. The level of play varied from 1 (recreational) to 5 (world-class). At the same time, most studies (33 of 36) reported results of level 3 (highly trained/national level) or higher (Table 1). Three articles did not report on the expertise level (Table 1). A total of 33 studies reported data collection/observation duration, with a median duration of 9 years and a range of 1–47 years (Table 1). In addition, 23 studies included male athletes only, 10 included female athletes only, and 6 included both male and female athletes (Table 1).

**Table 1:** Characteristics of the included studies

Study	Sport	Country	Level of play [28]	Duration of data collection	Number of included ACL injuries (female/male)	ACL injuries		
						Contact	Indirect	Non-contact
Johnston et al. [43]	American Football	USA	3–5	2013–2016	69 (0/69)	19	34	16
Schick et al. [26]	American Football	USA	3–5	2007–2016	53 (0/53)	20	11	22
Vargas et al. [59]	American Football, basketball, football, Australian Rules football, baseball, rugby	USA, ESP, AUS	3–5	2010–2017	26 (0/26)	N/A	0	26
Boden et al. [57]	American Football, basketball, football, netball	USA	2–5	N/A	23 (7/16)	8	N/A	15
Cochrane et al. [42]	Australian Rules football	AUS	3–5	1992–1998	34 (0/34)	11	4	19
Rolley et al. [45]	Australian Rules football	AUS	3–5	2016–2020	21 (21/0)	N/A	8	13
Axelrod et al. [46]	Basketball	USA	3–5	1997–2019	10 (10/0)	1	N/A	N/A
Gill et al. [47]	Basketball	USA	3–5	2006–2022	38 (0/38)	N/A	29	9
Krosshaug et al. [48]	Basketball	USA	2–5	N/A	39 (22/17)	4	7	28
Petway et al. [25]	Basketball	USA	4–5	1975–2022	35 (0/35)	9	21	5
Saito et al. [49]	Basketball	USA	4–5	2011–2022	27 (0/27)	0	11	15
Tosarelli et al. [50]	Basketball	EU	3–5	2013–2020	37 (0/37)	1	21	14
Heder Ternell et al. [51]	Basketball	FRA, GER, ITA, ESP, SWE	3–5	2018–2023	41(41/0)	0	23	18
Costello et al. [52]	Basketball	USA	3–5	2006–2022	31 (0/31)	2	17	12
Hurley et al. [53]	Basketball	USA	3–5	2009–2020	23 (0/23)	0	13	10
Sheehan et al. [58]	Basketball, football, American Football, handball	N/A	N/A	N/A	20 (13/7)	N/A	0	20
Boden et al. [56]	Basketball, handball, football, American Football, cheerleading, gymnastics	USA	3–4	1995–2007	29 (18/11)	N/A	8	21
Achenbach et al. [17]	Football	GER	4–5	2016–2017	37 (37/0)	6	14	17
Brophy et al. [32]	Football	N/A	1–4	N/A	55 (23/32)	31	N/A	N/A
D’Hooghe et al. [19]	Football	QAT, ITA	3–5	2014–2018	19 (0/19)	4	6	9
De Carli et al. [20]	Football	ITA, FRA, ESP, GER, UK	3–5	2010–2020	128 (0/128)	36	36	50
Della Villa et al. [21]	Football	ITA	3–5	2008–2018	134 (0/134)	16	59	59
Grassi et al. [33]	Football	Worldwide	N/A	1981–2015	34 (0/34)	12	7	15
Grassi et al. [34]	Football	ITA	3–5	N/A	21 (0/21)	N/A	5	16
Lucarno et al. [23]	Football	USA, GER, FRA, UK, ESP, ITA	3–5	2017–2020	35 (35/0)	4	12	19
Rekik et al. [35]	Football	QAT	3–4	2013–2019	15 (0/15)	3	4	8
Waldén et al. [36]	Football	EU	3–5	2001–2011	39 (0/39)	6	8	25
Buckthorpe et al. [37]	Football	Spain	3–5	2010–2022	115 (0/115)	16	49	50
Della Villa et al. [38]	Football	England	3–5	2010–2021	124 (0/124)	24	52	47
Ranzini et al. [39]	Football	UK, FRA, GER, ESP, International	3–5	2020–2023	27 (0/27)	N/A	9	18
Zago et al. [40]	Football	UK, FRA, GER, ESP, International	3–5	2020–2022	33 (33/0)	N/A	9	24
Gokeler et al. [41]	Football	Italy	3–5	2008–2018	47 (0/47)	N/A	N/A	47
Olsen et al. [24]	Handball	NOR	3–4	1988–2000	20 (20/0)	1	6	13
Koga et al. [30]	Handball, basketball	N/A	N/A	N/A	10 (10/0)	N/A	6	4
Akoto et al. [55]	Judo	EU	3–4	2010–2017	17 (8/9)	11	6	0
Belcher et al. [18]	Netball	AUS, NZL	3–4	2011–2019	21 (21/0)	N/A	7	14
Stuelcken et al. [54]	Netball	AUS, NZL	3–5	2008–2015	16 (16/0)	N/A	8	8
Della Villa et al. [22]	Rugby	Worldwide	3–5	2015–2019	57 (0/57)	18	15	24
Montgomery et al. [44]	Rugby	N/A	3–5	2014–2015	35 (0/35)	10	8	15

### Methodological Quality Characteristics

The quality of the studies was high (81–100%) in 2 studies, good (71–80%) in 17, moderate (60–70%) in 10, and low (< 60%) in 10 (Fig. 2, Table 2). QA-SIVAS scores ranged from 33 to 83%, with an average quality of 66% across all studies (Table 2). All articles clearly stated the study’s objectives. A total of 18 studies used video recordings from a representative sample, 8 provided sample information, and 4 specified video source/quality information. Detailed methodologies were described in 33 articles, and 35 reported systematic video analysis approaches (Table 2). Medical report information was included in 15 articles, while raters’ background/expertise was noted in 20. In 32 articles, multiple researchers evaluated the recordings. Three and two articles reported a control group and validated methods for quantitative biomechanical analysis, respectively. The main results were clearly presented in 38 articles, with clear injury case reporting in 37. The injury context was assessed in 37 articles, and 29 provided example screenshots or video frames. All articles discussed results in the context of current literature, with 36 addressing clinical/practical implications and all discussing study limitations.

**Table 2:** Risk of bias assessment using the Quality Appraisal for Sports Injury Video Analysis Studies (QA-SIVAS) tool of the included studies

	Objective stated	A representative sample was chosen	Information about sample is included	Information about video source and quality of the footage are included	Applied methods are described comprehensively	A systematic approach to video analysis was chosen	Medical report information are included	Background/expertise of raters is stated	Findings are observed by more than one researcher	A control group is included	A quantitative biomechanical analysis was conducted using validated methods	The main results of the study are clearly described	Absolute numbers or proportions of injury cases for each/the main outcome are reported	Details about the injury context are included	Example screenshots/video frames are included	Findings are discussed within the context of the current evidence	Clinical/practical implications of the results are discussed	Limitations of the study are addressed	Score (%)
Achenbach et al. [17]	1	1	1	0	1	1	1	1	1	0	0	1	1	1	1	1	1	1	83
Akoto et al. [55]	1	0	0	0	1	1	1	1	1	0	0	1	1	1	1	1	1	1	72
Axelrod et al. [46]	1	0	1	0	0	0	0	0	1	0	0	1	1	1	0	1	0	1	44
Belcher et al. [18]	1	0	0	0	1	1	0	1	1	0	0	1	1	1	1	1	1	1	67
Boden et al. [57]	1	0	0	0	0	0	0	0	0	0	0	0	1	1	0	1	1	1	33
Boden et al. [56]	1	0	0	0	1	1	0	0	0	1	0	1	0	1	1	1	0	1	50
Brophy et al. [32]	1	0	0	0	1	1	0	0	1	0	0	1	1	1	1	1	1	1	61
Buckthorpe et al. [37]	1	1	0	0	1	1	0	1	1	0	0	1	1	1	1	1	1	1	72
Cochrane et al. [42]	1	0	0	1	1	1	0	0	1	0	0	1	1	1	0	1	1	1	61
Costello et al. [52]	1	1	1	0	0	0	0	0	1	0	0	1	1	1	0	1	1	1	56
D'Hooghe et al. [19]	1	0	1	0	1	1	1	0	1	0	0	1	1	1	1	1	1	1	72
De Carli et al. [20]	1	0	0	0	1	1	0	0	1	0	0	1	1	1	0	1	1	1	56
Della Villa et al. [21]	1	1	0	0	1	1	1	1	1	0	0	1	1	1	1	1	1	1	78
Della Villa et al. [22]	1	1	0	0	1	1	1	1	1	0	0	1	1	1	1	1	1	1	78
Della Villa et al. [38]	1	1	0	0	1	1	0	1	1	0	0	1	1	1	1	1	1	1	72
Gill et al. [47]	1	1	0	0	1	1	1	1	1	0	1	1	1	1	0	1	1	1	78
Gokeler et al. [41]	1	1	0	0	1	1	1	1	1	0	0	1	1	1	1	1	1	1	78
Grassi et al. [33]	1	0	0	0	1	1	0	0	1	0	0	1	1	1	1	1	1	1	61
Grassi et al. [34]	1	0	0	0	1	1	0	0	1	0	0	1	1	1	1	1	1	1	61
Heder Ternell et al. [51]	1	1	1	0	1	1	0	1	1	0	0	1	1	1	1	1	1	1	78
Hurley et al. [53]	1	1	0	0	0	1	0	0	1	0	0	1	1	1	0	1	1	1	56
Johnston et al. [43]	1	1	0	0	1	1	0	1	1	0	0	1	1	1	0	1	1	1	67
Koga et al. [30]	1	0	0	1	1	1	0	0	0	0	1	1	1	1	1	1	0	1	61
Krosshaug et al. [48]	1	0	0	1	1	1	0	1	1	0	0	1	1	1	1	1	1	1	72
Lucarno et al. [23]	1	1	0	0	1	1	1	1	1	0	0	1	1	1	1	1	1	1	78
Montgomery et al. [44]	1	1	0	0	1	1	0	1	1	1	0	1	1	1	1	1	1	1	78
Olsen et al. [24]	1	0	0	0	1	1	1	1	1	0	0	1	1	1	1	1	1	1	72
Petway et al. [25]	1	0	0	0	0	0	0	1	0	0	0	1	1	0	1	1	1	1	44
Ranzini et al. [39]	1	0	0	0	1	1	1	0	1	0	0	1	1	1	1	1	1	1	67
Rekik et al. [35]	1	1	0	0	1	1	1	1	1	0	0	1	1	1	1	1	1	1	78
Rolley et al. [45]	1	1	0	0	1	1	0	0	0	0	0	1	1	1	0	1	1	1	56
Saito et al. [49]	1	0	1	0	1	1	0	0	0	0	0	1	1	1	1	1	1	1	61
Schick et al. [26]	1	0	0	0	0	1	0	0	1	0	0	1	1	1	0	1	1	1	50
Sheehan et al. [58]	1	0	0	0	1	1	1	0	0	1	0	1	0	0	1	1	1	1	56
Stuelcken et al. [54]	1	1	0	0	1	1	0	1	1	0	0	1	1	1	1	1	1	1	72
Toserelli et al. [50]	1	0	1	0	1	1	1	1	1	0	0	1	1	1	1	1	1	1	78
Vargas et al. [59]	1	0	0	0	1	1	0	0	1	0	0	1	1	1	1	1	1	1	61
Waldén et al. [36]	1	1	0	1	1	1	1	1	1	0	0	1	1	1	1	1	1	1	83
Zago et al. [40]	1	1	1	0	1	1	1	0	1	0	0	1	1	1	1	1	1	1	78
Sum (%)	100	46	21	10	85	90	38	51	82	8	5	97	95	95	74	100	92	100	66

### Injury Characteristics

Collectively, the 39 primary studies investigated 1551 video-recorded ACL injuries. In addition, 20.8% of the investigated injuries in the studies were sustained by women, while 79.2% were sustained by men. Most injuries were classified as noncontact (*n* = 745), followed by indirect contact (*n* = 533) and direct contact (*n* = 273; Table 3). The precise relative shares among injury classifications could not be calculated, as some studies only reported pooled injury counts (e.g., for indirect plus noncontact injuries). Moderator analysis revealed a significant impact of the type of sport on direct contact proportions (*p* < 0.05; Table 3), which were more common in judo (64.7%; number of studies (*n*) = 1) than in COF (32.1%; 95% CI 26.4–37.9%; *n* = 5), football (22.5%; 95% CI 15.6–30.3%; *n* = 11), basketball (6.1%; 95% CI 1.7–12.8%; *n* = 8), or handball (5%; *n* = 1). Indirect contact ACL injury proportions were statistically significantly moderated by the type of sports, with 48.9% in basketball (95% CI 36.7–61.2; *n* = 7), showing higher rates than in judo (35.3%; *n* = 1), football 35.0% (95% CI 29.6–40.7%; *n* = 10), handball 30% (*n* = 1), or COF 26.8% (95% CI 15.4–40.0%; *n* = 5). The type of sport was not significantly associated with the occurrence of noncontact injuries (*p* > 0.05).

**Table 3:** Proportions of noncontact, indirect contact, and direct contact ACL injuries

	Estimated proportion (%)	*p*	95% CI (%)	*df*	*I*^2^ (%)	Moderator *p* value	*n*
Non contact ACL injuries	44.0	0.038	(38.5–49.7)	24	72.9	0.152	24
Indirect contact ACL injuries	36.4	< 0.001	(31.1–41.8)	23	71.6	0.017	24
Direct contact ACL injuries	19.5	< 0.001	(14.2–25.5)	26	84.2	< 0.001	26
Non contact and indirect contact ACL injuries pooled	80.5	< 0.001	(74.5–85.8)	26	84.2	< 0.001	26
Indirect contact and contact ACL injuries pooled	56.7	0.020	(51.0–62.3)	23	72.6	0.152	24

### Movement Patterns

When combining all ACL injuries (noncontact, indirect contact, and direct contact), the highest proportions occurred during ball actions (45.7%), pressing/tackling (40.9%), and cutting (36.6%, Table 4). The moderator analysis yielded significant effects for landing in general (*p* < 0.05). ACL injuries during landing (single and double leg landing combined) were more frequent in netball (81.2%, *n* = 2) than in basketball (32.5%; 95% CI 21.8–44.3%; *n* = 9), handball (20.0%; *n* = 1), COF (16.9%; 95% CI 5.9–39.5%; *n* = 5), or football (12.8%; 95% CI: 7.3–19.6%; *n* = 9). The moderator analysis yielded significant effects for pressing/tackling situations (*p* < 0.05). Injuries in soccer (44.3%; 95% CI 39.7–48.8%; *n* = 13) were more frequent than in COF (17.5%; *n* = 2).

**Table 4:** Combined proportions of all ACL injuries, noncontact, and indirect contact, as well as direct contact and indirect contact ACL injuries analyzed per movement pattern

	Estimated proportion (%)	*p* value	95% CI (%)	*df*	*I*^2^ (%)	Moderator *p* value	n
Movement patterns/players action							
*All ACL injuries pooled*							
Landing	23.4	< 0.001	(17.5–29.8)	29	83.4	0.016	25
Single-leg landing	21.7	< 0.001	(14.0–30.5)	15	76.9	0.287	11
Double-leg landing	9.9	< 0.001	(3.1–20.0)	13	87.8	0.142	10
Cutting	36.6	0.015	(26.5–47.4)	23	90.8	0.896	21
Deceleration	31.0	0.006	(18.9–44.6)	14	91.0	0.313	11
Acceleration	10.4	< 0.001	(2.6–22.5)	4	82.2	0.355	4
Ball action	45.7	< 0.001	(31.0–60.9)	15	93.8	0.099	15
Being tackled	20.1	< 0.001	(15.4–25.2)	13	62.7	0.075	14
Kicking	9.3	< 0.001	(4.3–15.9)	4	51.2	0.345	5
Heading	7.4	< 0.001	(3.0–13.6)	3	27.3		
Passing	13.8	< 0.001	(3.1–30.6)	5	90.3	0.348	6
Pressing/ Tackling	40.9	< 0.001	(34.8–47.1)	14	66.6	0.002	15
Ball protection	9.6	< 0.001	(6.2–13.5)	5	0.0	0.953	4
*Non-contact and indirect contact ACL injuries pooled*							
Landing	23.8	< 0.001	(17.6–30.6)	28	83.1	0.036	25
Single-leg landing	20.8	< 0.001	(13.6–29.0)	12	64.6	0.763	10
Double-leg landing	8.9	< 0.001	(2.6–18.6)	10	82.5	0.114	8
Cutting	38.0	< 0.001	(25.3–51.6)	22	93.2	0.360	20
Deceleration	28.8	0.012	(14.8–45.2)	11	88.1	0.212	10
Ball action	34.8	< 0.001	(22.6–48.2)	16	92.0	0.004	15
Being tackled	21.1	< 0.001	(17.9–24.5)	12	55.2	0.101	13
Pressing/ Tackling	41.8	0.017	(35.2–48.5)	13	61.2	0.002	14
*Indirect contact and contact ACL injuries pooled*							
Landing	22.8	0.007	(8.1–42.2)	12	80.1	0.223	9
Single-leg landing	18.3	< 0.001	(6.6–34.2)	8	66.1	0.420	6
Double-leg landing	5.8	0.076	(1.5–12.7)	7	22.8	0.175	5
Cutting	12.7	< 0.001	(4.8–23.6)	14	72.3	0.176	12
Deceleration	12.5	< 0.001	(2.8–27.6)	8	74.2	0.355	7
Ball action	23.1	0.044	(4.9–49.2)	5	87.2	0.185	5
Being tackled	37.2	0.048	(25.4–49.9)	10	80.8	0.856	11
Pressing/ Tackling	22.3	< 0.001	(13.9–32.1)	13	77.6	0.795	14

Noncontact injuries were most frequent during cutting (53.8%), followed by pressing/tackling (50.2%), decelerating (38.9%), and landing (30.1%, Table 5). The moderator analysis revealed that noncontact ACL injuries during pressing/tackling and deceleration situations were significantly affected by the type of sports (*p* < 0.05). For pressing/tackling situations, higher frequencies were reported in soccer (55.8%; 95% CI 49.8–61.8%; *n* = 9) than in COF, where one study [44] explicitly reported zero noncontact injuries. Noncontact injuries during deceleration were more frequent in netball (58.0%, *n* = 2) than in football (35.3%, *n* = 1), COF (8.6%, *n* = 2), or handball (7.7%; *n* = 1). No association was detected between the type of sport and ACL injury during other movement patterns (*p* > 0.05).

**Table 5:** Proportions of noncontact, indirect contact, and direct contact ACL injuries for movement pattern, movement speed, and game situation

	Estimated proportion (%)	*p* value	95%CI (%)	*df*	*I*^2^ (%)	Moderator *p* value	*n*
Movement patterns/player’s action							
*Non-contact ACL injuries*							
Landing	30.1	0.048	(18.0–43.8)	13	79.0	0.442	10
Single-leg landing	19.7	< 0.001	(10.2–31.5)	7	51.9	0.302	5
Double-leg landing	8.8	< 0.001	(0.3–27.1)	7	84.3	0.240	4
Cutting	53.8	< 0.001	(40.4–67.0)	13	74.8	0.132	11
Deceleration	38.9	< 0.001	(18.4–61.2)	7	85.0	0.004	6
Ball possesion	23.4	< 0.001	(11.9–37.4)	4	46.8	0.096	4
Being tackled	1.7	< 0.001	(0.4–3.9)	8	0	0.965	9
Pressing/tackling	50.2	< 0.001	(37.7–62.6)	9	75.9	0.001	10
*Indirect contact ACL injuries*							
Landing	30.0	< 0.001	(12.3–51.6)	11	73.6	0.288	9
Single-leg landing	25.7	0.006	(11.8–42.7)	7	50.0	0.416	6
Double-leg landing	8.3	< 0.001	(2.5–17.3)	6	17.9	0.187	5
Cutting	22.5	0.003	(8.7–40.5)	12	73.5	0.199	11
Deceleration	25.5	0.040	(7.9–48.9)	6	63.5	0.567	6
Ball possesion	44.8	0.003	(22.5–68.3)	4	64.1	0.174	4
Being tackled	56.1	< 0.001	(39.8–71.6)	8	73.0	0.029	9
Pressing/tackling	24.8	< 0.001	(12.8–39.3)	8	70.5	0.537	9
*Contact ACL injuries*							
Being tackled	23.9	0.031	(6.6–47.6)	7	85.3	0.629	8
Pressing/tackling	24.2	0.011	(9.1–43.7)	10	83.6	0.855	11
Movement Speed							
*Non-contact ACL injuries*							
Horizontal speed high	70.7	< 0.001	(59.6–80.6)	4	2.3	0.441	5
Horizontal speed low	20.9	< 0.001	(11.5–32.2)	3	0.0		
Horizontal speed zero	4.4	< 0.001	(0.3–13.0)	2	0.0		
*Indirect contact ACL injuries*							
Horizontal speed high	36.1	0.012	(6.3–74.0)	4	81.9	0.325	5
Horizontal speed low	9.1	0.036	(0.7–25.3)	3	23.8		
Horizontal speed zero	7.1	0.317	(0.3–21.4)	2	0.0		
Vertical speed zero	50.8	0.041	(2.5–98.1)	2	89.4	0.225	3
Game situations							
*Non-contact ACL injuries*							
Offensive	58.5	< 0.001	(35.7–79.6)	5	84.7	0.376	5
Defensive	37.6	0.004	(16.3–61.8)	5	86.2	0.476	5
*Indirect contact ACL injuries*							
Offensive	22.8	0.081	(3.1–53.7)	5	64.9		
Defensive	42.7	0.007	(8.0–77.9)	5	72.5		
*Contact ACL injuries*							
Offensive	52.9	< 0.001	(43.2–62.4)	6	0.0	0.175	7
Defensive	46.1	< 0.001	(36.6–55.7)	6	0.0	0.089	7

Indirect contact injuries were most frequent while being tackled (56.1%), followed by situations with ball possession (44.8%) and landing (single- and double-leg landing combined, 30.0%, Table 5). For injuries occurring while being tackled, the moderator analysis revealed a significant effect of the type of sport, with higher frequencies reported in COF (100%; eight indirect injuries, all of which occurred were while being tackled; *n* = 1 [44]) than in soccer (49.1%, 95% CI 36.1–62.3%; *n* = 8). No association was detected between the type of sport and any other movement pattern for indirect contact injuries.

For contact ACL injuries, injury frequencies while being tackled (23.9%) were similar to those during pressing/tackling (24.2%, Table 5) situations. Moderator analysis revealed no differences between sports. The supplemental digital content provides pooled estimates for combinations of direct and indirect contact as well as indirect and noncontact injuries.

### Movement Velocity

With all injuries combined, the highest summary proportions were observed for situations with high (53.8%) and low horizontal velocity (34.8%), while injuries at no velocity (6.2%) were scarce (Table 6). For vertical velocity, an opposite pattern was observed, with a higher proportion of injuries sustained at no vertical (63.5%) versus low (19.0%) or high vertical velocity (11.5%). Moderator analyses revealed a significant impact of sports (*p* < 0.05): Injuries with high vertical velocity were highest for netball (75.0%; *n* = 1) but comparably low for basketball (19.6%; *n* = 2), COF (6.4%; 95% CI 3.0–10.9%; *n* = 3), or football (6.0%; 95% CI 4.0–8.4%; *n* = 5).

**Table 6:** Combined proportions of all ACL injuries, noncontact, and indirect contact, as well as direct contact and indirect contact ACL injuries analyzed per movement speed

	Estimated proportion (%)	*p*	95% CI (%)	*df*	*I*^2^ (%)	Moderator *p* value	*n*
Movement Speed							
*All ACL injuries pooled*							
Horizontal speed high	53.8	< 0.001	(43.6–63.9)	14	88.7	0.696	15
Horizontal speed low	34.8	0.007	(24.5–45.9)	15	91.1	0.627	16
Horizontal speed zero	6.2	< 0.001	(4.3–8.4)	12	29.0	0.298	13
Vertical speed high	11.5	< 0.001	(6.3–18.1)	10	81.5	0.017	11
Vertical speed low	19.0	< 0.001	(10.3–29.6)	9	89.6	0.145	10
Vertical speed zero	63.5	0.029	(51.4–74.8)	10	89.7	0.057	11
*Non-contact and indirect contact ACL injuries pooled*							
Horizontal speed high	53.0	< 0.001	(37.8–67.9)	9	88.5	0.874	10
Horizontal speed low	41.6	< 0.001	(27.6–56.3)	10	88.7	0.291	11
Horizontal speed zero	5.1	< 0.001	(3.1–7.6)	8	0.0	0.087	9
Vertical speed high	17.1	< 0.001	(7.3–30.0)	7	87.3	0.071	8
Vertical speed low	25.9	0.001	(13.4–40.8)	5	85.9	0.044	6
Vertical speed zero	56.4	< 0.001	(39.1–72.9)	6	89.2	0.084	7
*Indirect contact and contact ACL injuries pooled*							
Horizontal speed high	28.3	0.038	(2.4–67.6)	4	90.1	0.330	5
Horizontal speed low	7.1	< 0.001	(0.4–20.8)	4	53.1	0.331	4
Horizontal speed zero	6.3	< 0.001	(0.0–22.3)	3	49.7		
Vertical speed zero	52.1	0.957	(0.2–99.9)	2	95.1		

When considering noncontact injuries only, most injuries occurred at high horizontal velocity (70.7%). Indirect injuries also occurred most often at high horizontal velocity, although the proportion was lower (36.1%, Table 5). No pooling was possible for direct contact injuries.

Moderator analysis for noncontact and indirect contact injuries did not reveal any statistically significant effects. No moderator analysis was possible for the remaining movement speeds, or for noncontact, indirect, or direct contact injuries owing to insufficient data.

### Game Situation

Considering all injuries, the highest ACL injury proportions were reported for situations with ball possession (67.5%) and offensive game situations (55.4%, Table 7). Concerning the time point of injury, the distributions across quarters were relatively similar (Table 7). Moderator analyses revealed no impact of the type of sport with regards to ball possession and tactical situation (*p* > 0.05) but an impact in terms of the time point of injury. The highest number of injuries during the first quarter were sustained in netball (37.8%; *n* = 1) and football (37.5%; *n* = 2), followed by COF (25.9%, 95% CI 15.4–41.8%; *n* = 3) and basketball (16.0%; *n* = 2).

**Table 7:** Combined proportions of all ACL injuries, noncontact, and indirect contact, as well as direct contact and indirect contact ACL injuries analyzed per game situations

	Estimated proportion (%)	*p*	95% CI (%)	*df*	*I*^2^ (%)	Moderator *p* value	*n*
Game situations							
*All ACL injuries pooled*							
Offensive	55.4	< 0.001	(46.6–64.1)	25	88.9	0.137	24
Defensive	40.4	0.037	(31.7–49.4)	25	89.5	0.062	24
Quarter 1	28.8	< 0.001	(21.8–35.8)	8	56.3	0.019	8
Quarter 2	24.8	< 0.001	(16.4–33.1)	8	76.1	0.074	8
Quarter 3	20.3	< 0.001	(13.1–27.5)	8	70.8	0.086	8
Quarter 4	29.0	0.002	(10.7–47.3)	8	96.3	0.261	8
Ball possesion	67.5	< 0.001	(52.7–80.6)	9	90.2	0.292	9
*Non-contact and indirect contact ACL injuries pooled*							
Offensive	51.7	< 0.001	(41.4–60.9)	17	83.7	0.059	17
Defensive	38.1	< 0.001	(26.9–50.0)	18	89.8	0.030	17
Quarter 1	26.4	< 0.001	(18.3–34.5)	7	38.3	0.021	7
Quarter 2	26.7	< 0.001	(14.9–38.4)	7	76.5	0.084	7
Quarter 3	15.2	< 0.001	(6.9–23.5)	7	67.9	0.326	7
Quarter 4	32.8	0.006	(9.5–56.2)	7	95.5	0.399	7
Ball possesion	47.0	< 0.001	(29.8–64.6)	8	76.7	0.277	6
*Indirect contact and contact ACL injuries pooled*							
Offensive	24.9	< 0.001	(15.2–36.2)	8	72.8	0.626	8
Defensive	20.5	< 0.001	(13.4–28.9)	8	56.5	0.252	8
Quarter 1	26.6	< 0.001	(15.4–37.8)	2	0.0		
Quarter 2	17.7	< 0.001	(8.0–27.3)	2	0.0		
Quarter 3	14.1	0.013	(3.0–25.3)	2	26.2		
Quarter 4	12.9	0.111	(0.0–28.7)	2	61.1		

Noncontact injuries (58.5%) mainly occurred during offensive play, while indirect contact injuries had the highest frequency during defensive play (42.7%; Table 5). Pooling of estimates was either not possible or revealed no significant effect.

## Discussion

This study is the first meta-analysis to systematically synthesize video-based evidence on anterior cruciate ligament (ACL) injury situations across sports. We included 39 studies comprising 1551 video-documented ACL injuries, with football, basketball, and COF (American Football, Australian Rules football, rugby) contributing the majority of cases, alongside netball, handball, and judo. By pooling and comparing data across these diverse contexts, the present work provides the most comprehensive quantitative overview to date of video-analyzed movement patterns and game situations associated with ACL injuries. Understanding these patterns is of critical importance, as they reveal the situational contexts in which ACL loading and subsequent rupture most often occur, thereby offering essential guidance for sport-specific surveillance and the design of targeted prevention strategies [8, 60].

### Contact Mechanisms

In our video-based meta-analysis of predominantly team sports, noncontact mechanisms accounted for 44% of ACL injuries, indirect contact for 36%, and direct contact for 20%. Moderator analyses indicated that the distribution of injury mechanisms varied by sport. Across team sports, direct‑contact injuries were most frequent in COF (32.1% [95% CI 26.4–37.9], *n* = 5), intermediate in football (22.5% [15.6–30.3], *n* = 11), and uncommon in basketball (6.1% [1.7–12.8], *n* = 8). By contrast, indirect‑contact injuries comprised nearly half of basketball ACL injuries (48.9% [36.7–61.2], *n* = 7) but a smaller fraction in football (35.0% [29.6–40.7], *n* = 10) and COF (26.8% [15.4–40.0], *n* = 5); noncontact proportions did not differ significantly between sports. Smaller samples in other sports showed specific extremes (e.g., judo skewed toward direct contact, with 64.7%, whereas netball presented low direct contact involvement). These sport-specific patterns align with the most recent systematic review, which reported that basketball is dominated by indirect contact mechanisms (~ 60%), football is primarily noncontact (~ 47%), and American Football/rugby have larger contact contributions [13]. However, this review synthesized video data together with athlete/medical staff reports and did not meta-analyze proportions, which may explain the differences in magnitude [13].

### Movement Patterns

In our team-sports-dominated sample of studies, noncontact ACL injuries most frequently occurred during cutting (53.8%), followed by pressing/tackling (50.2%), decelerating (38.9%), and landing (30.1%). Indirect contact mechanisms were most frequently associated with being tackled (56.1%), followed by situations with ball possession (44.8%) and landing (30.0%). For direct contact injuries, pooled action-specific estimates were available for being tackled (23.9%) and pressing/tackling (24.2%). Moderator analyses indicated sport-specific differences for tackling-related patterns. Pressing/tackling was more frequent in soccer than in COF when pooling all injuries and when restricting analyses to noncontact cases. For indirect contact injuries, the proportion of cases classified as being tackled differed between sports (higher in COF than in soccer), although this finding should be interpreted cautiously owing to the limited number of eligible studies in some sports. No significant moderator effects were observed for direct contact injury patterns, but action-specific data were sparse. Notably, tackling-related situations showed sport-dependent patterns. In soccer, pressing/tackling accounted for a substantial proportion of injuries, including in noncontact cases, which may reflect high-demand defensive actions (e.g., pressing or attempted tackles) that do not necessarily involve knee-directed contact. In contrast, indirect contact injuries in COF were frequently classified as being tackled, suggesting that perturbations and body–contact dynamics during tackles may be a more dominant feature in these codes. Given the small number of eligible studies in some sports and the conceptual overlap between “pressing” and “tackling,” these findings should be interpreted with caution and motivate more granular, consensus-driven reporting (e.g., separating pressing from tackling and specifying whether contact occurred, where, and when, relative to the injury).

Overall, these findings confirm that cutting, pressing/tackling, deceleration, and landing are the key high-risk movement contexts when no contact with opponents is involved, whereas being tackled and pressing/tackling are the most common contact-related scenarios. Our findings corroborate those of Sunderg et al. [13], who, based on both video- and non-video sources, also identified cutting and landing as key mechanisms underlying noncontact ACL injuries in team sports. However, by incorporating more recent, larger-sample studies [27, 38], we identified pressing/tackling and being tackled as frequently occurring movement patterns, particularly in football and COF, which might have previously not been recognized.

### Movement Velocity

Across the pooled team-sport sample, ACL injuries occurred most frequently at high horizontal velocity (53.8%), with additional contributions from low horizontal velocity (34.8%) and no horizontal motion (6.2%). The majority of injuries were sustained with no vertical component (63.5%), while fewer occurred at low (19.0%) or high (11.5%) vertical velocity. These distributions suggest that both rapid horizontal movements and vertical loading scenarios, particularly landings, are important contexts for ACL injury. Previous work [13] has emphasized the relevance of high-horizontal speed play in several sports; our pooled estimates extend this by demonstrating that noncontact injuries can also arise in stationary or low-horizontal-velocity situations, potentially due to high vertical ground reaction force impulses. However, the assumption that noncontact, low-horizontal-velocity ACL injuries were due to high vertical velocity remains speculative at the moment, because the included studies rarely reported the results in a way that would allow this assumption to be directly checked. Future studies should report results in a way (ideally on a per-injury basis) that allows for a joint interpretation of movement speed and contact mechanism, as well as situational patterns to clarify under which precise conditions contact, indirect, and noncontact ACL injuries occur.

### Game Situations

When considering the broader game context, 67.5% of ACL injuries occurred during ball possession and 55.4% during offensive play. Noncontact injuries showed a predominance in offensive phases (58.5%), whereas indirect contact injuries had a comparatively larger share in defensive phases (42.7%). Moderator analyses of injury timing indicated that time-of-match distributions differed by sport: Injuries clustered in the first quarter in football (37.5%; *n* = 2) and netball (37.8%; *n* = 1) were closer to a uniform distribution in contact‑oriented football (COF; 25.9%, 95% CI 15.4–41.8%; *n* = 3), and were less frequent early in basketball (16.0%; *n* = 2). Because a “first quarter” represents roughly 25% of total match time, the football and netball figures suggest over‑representation of early phase injuries, whereas basketball shows under‑representation. These differences may reflect sport-specific opening-phase demands, warm-up strategies, or distinct match structures (e.g., substitution patterns, stoppages, and set-play density in basketball and COF).

### Practical Implications

Injury prevention strategies in sports should be mechanism‑specific, with movement scenarios and game situations refining where the primary leverage lies (rules/policy, coaching and technique, physical preparation, or equipment) [8]. In the sections that follow, we aim to derive the potentially most effective prevention strategies for each sport from our findings. We derived prevention strategies for football, basketball, and COF, as the findings for these sports were likely most robust, given that they are based on the largest number of included studies in our analysis.

For football, noncontact mechanisms were the predominant driver of ACL injuries, with the highest noncontact frequencies observed during cutting (53.8%), pressing/tackling actions (50.2%), decelerating (38.9%), and landing (30.1%). Accordingly, preventive work in football should incorporate scenario-based training that refines and enhances cutting [61], braking [62], and single- and double-leg landing techniques [63, 64]. Given the energy absorption requirements of the identified high-risk movement patterns, these drills should be coupled with the development of sufficient eccentric strength capacities in the muscle groups involved in these tasks [65]. Because injuries frequently occurred during ball possession (67.5%), and noncontact cases were more common during offensive play (58.5%), these drills should routinely include the ball and neurocognitive decision-making (i.e., executive function) demands to promote automatic control under realistic game conditions [66].

Indirect‑contact injuries were also common in football (35.0%), most often while being tackled (56.1%), with additional contributions from landing (single‑ and double‑leg landing combined 30.0%) and single‑leg landing (25.7%). To address these situations, we recommend integrating perturbation elements into football-specific cutting and landing tasks (e.g., controlled pushes or band pulls while changing direction or receiving the ball), with explicit emphasis on trunk control, single-leg stability, and rapid recovery of alignment when destabilized. Embedding these perturbations within ball‑in‑possession drills (also to emphasize an external focus of attention) reflects the contexts in which indirect‑contact injuries frequently arise. In an ACL injury context, perturbation training has been mainly applied during RTS settings [67]. However, for preventive approaches, perturbations may need to be adjusted in intensity to induce the desired prevention effects.

Although direct‑contact injuries represent a smaller share in football (22.5%), they warrant targeted countermeasures. From a policy and coaching perspective, this includes stricter sanctioning and consistent officiating for knee-directed challenges (e.g., late or low tackles into a planted limb or knee-to-knee collisions), alongside technical coaching on safer entry and exit from duels (reducing planted-leg exposure and preparing to yield or redirect contact). Finally, moderator analyses also indicated an early match clustering in football, with 37.5% of injuries occurring in the first 25% of the game. This counterintuitive pattern (unlikely to be fatigue-driven) suggests re-examining pre-kick-off and on-field activation, e.g., keeping the warm-up closer to kick-off and adding brief, game-speed cut/deceleration/landing sequences. Future work should confirm this with time-stamped exposure data in relation to descriptions of warm-up strategies.

In basketball, direct‑contact mechanisms were uncommon (6.1%), whereas indirect‑contact injuries were the largest share across sports (48.9%). High horizontal speed was relatively infrequent (19.6%), and landing featured prominently among movement patterns (32.5%), underscoring the potential impact of more vertical‑impulse related contexts. Because indirect‑contact events predominate, incorporating perturbation elements into jump‑landing and cutting drills (e.g., controlled shoulder bumps, elastic‑band pulls, aerial/body contact at or just after landing), with explicit training of trunk control, single‑leg stability, and rapid recovery of alignment when destabilized, could be a valuable strategy for ACL injury prevention in basketball. Coupling these drills with ball-in-hand scenarios to mirror the contexts in which indirect perturbations more generally arise may further increase their effectiveness.

As in other sports, the prevention of noncontact injuries should emphasize jump- and land-specific technique (single and double leg), coached at game speed and under visual-attention demands, with eccentric strength work to support controlled decelerations on take-off and landing. Given the overall prominence of ball possession and offensive phases in the pooled data, we recommend integrating ball handling and passing tasks to foster an external focus of attention while maintaining safe knee alignment under realistic decision‑making. Given the low frequency of direct‑contact ACL injuries in basketball, rule‑based levers are likely limited. No early match clustering was evident in basketball (16.0% in the first quarter), providing no immediate signal to modify pre-tip-off warm-up timing.

In COF, direct‑contact mechanisms accounted for the highest share among the team sports analyzed (32.1%). Accordingly, prevention might prioritize tackle-related exposures: refine tackle technique (minimize knee-directed impact to a planted limb), strengthen officiating and sanctioning for knee contact, and consider equipment strategies (e.g., task-adaptive knee bracing) where feasible. Precedents in these codes (e.g., recent tackle‑law initiatives in rugby to reduce concussion risk [68]) illustrate how targeted rule adjustments might shift behavior; however, changes must balance player safety with the fabric of the game, and their effects should be monitored for actual prevention effects and unintended consequences [69]. Indirect and noncontact injuries also contributed meaningfully to ACL injuries in COF. Consequently, the movement- and context-focused measures outlined for football and basketball (cutting/braking and landing technique at high speed, coupled with adequate eccentric strength and decision-making demands) are directly applicable here and should be incorporated alongside contact-focused measures.

Evidence for netball derives from only two studies, so estimates carry substantial uncertainty. Within this limited sample, landing (single and double leg) accounted for 81.2% of ACL injuries, and noncontact deceleration was comparatively frequent (58.0%), indicating that prevention should prioritize jump and landing technique (single and double leg) and high‑quality deceleration into landings at match speed, supported by adequate eccentric braking capacity. In one study that reported horizontal velocity, injuries were often preceded by high horizontal velocity (81.3%), suggesting that drills rehearsing rapid approach-deceleration sequences may be beneficial [54]. Given that indirect‑contact events can still arise in congested spaces, it is reasonable also to include perturbations during landing and ball‑handling tasks to train rapid recovery of alignment. Finally, moderator analyses indicated early match clustering (37.8% in the first quarter). While the causal drivers are unclear, aligning warm-up/field activation with brief, game-speed landing/deceleration exposures closer to the start may be prudent.

### Limitations

Some methodological aspects warrant consideration. The included studies showed significant variability in the analyzed sports and methodologies. This finding highlights the need for standardized definitions and reporting methods, which would benefit future research. We believe that the 18 items of the recently developed QA-SIVAS scale [11] could be a guideline for these methodological improvements.

The quality assessment of the included studies in this review, using the QA-SIVAS scale, revealed a broad spectrum of methodological rigor, with scores ranging from 33 to 83%. Two studies were classified as high quality (81–100%), while the majority fell within the good (71–80%) and moderate (60–70%) categories. However, ten studies were deemed to have low quality (< 60%). Certain aspects of the studies were consistently well reported, including the objectives, methodological descriptions, main results, injury context, and discussions within the current evidence base. However, there was substantial room for improvement in areas such as representative sample recruitment, reporting information about the sample, video source/quality, medical report information, and descriptions of the raters’ backgrounds. These findings underscore the importance of applying quality criteria more stringently in future research to ensure the reliability and validity of video analysis studies in sports injury research. This includes the necessity for consistent reporting standards of injury mechanisms (i.e., contact, indirect contact, noncontact injuries) and the use of standardized definitions and terms for movement situations and patterns, at least for specific sports. A further limitation concerns the granularity with which defensive actions were described in the primary studies. For meta-analytic pooling across sports, we used harmonized categories (e.g., “pressing/tackling” and “being tackled”); however, “tackling” can encompass mechanistically distinct scenarios that likely differ between codes (e.g., noncontact pressing or attempted tackles in soccer versus body-contact tackles in COF, and injuries sustained by the tackler, such as when the lower limb becomes pinned, versus injuries to the ball carrier). Although we revisited the original papers to determine whether a more fine-grained classification (e.g., pressing versus tackling and contact versus noncontact tackling) was feasible, most studies did not provide injury-level descriptors with sufficient detail (player role, tackle type, and whether/where/when contact occurred relative to the injury). Consequently, some misclassification across categories cannot be excluded, and pooled estimates for these defensive actions should be interpreted with caution. Future video-analysis studies would benefit from consensus-driven reporting that explicitly separates pressing from tackling and documents, on a per-injury basis, the presence, location, and timing of contact and whether the injured athlete was the tackler or the player being tackled, to improve comparability and synthesis across sports. More broadly, this illustrates that comparability across sports will depend on more explicit operational definitions and reporting of whether contact occurred, where it occurred, and when it occurred relative to the injury event, alongside clearer separation of defensive actions. To improve clarity, future studies should, e.g., differentiate contact on the basis of (1) force magnitude and direction, distinguishing minor interactions from those significantly altering postural stability or knee-joint loading; (2) timing, as contact occurring several strides before the injury may differ biomechanically from contact immediately preceding or during the injurious movement; and (3) biomechanical consequences, such as contact-induced balance shifts or increased valgus loading. Standardizing these classifications through predefined criteria or biomechanical thresholds will enhance comparability across studies and sports, ultimately refining injury prevention strategies. Owing to the different classifications and descriptions of movements and situations, meta-analytical approaches and comparative studies are challenging to implement. Initial approaches to sport-specific categorization of injury-inciting events have recently been developed for football and netball [70, 71].

Another limitation of this study was that our moderator analysis for noncontact ACL injuries in different sport-specific situations was hindered by the small number of eligible studies, which prevented us from drawing definitive conclusions. Hopefully, this limitation can be mitigated by future high-quality studies that differentially assess the situational circumstances leading to ACL injuries in sports other than football.

The overall lower number of injury cases in female athletes and studies only reporting on one sex is a limitation that needs to be addressed in the current sex data gap in sports medicine research [72]. As injury rates in team sports [73] and certain risk factors [74] differ between sexes, we call for the collection of data for both female and male athletes in the future to provide sex-specific injury risk reduction programs [75]. Furthermore, video analysis studies were almost entirely conducted on samples of highly trained athletes (Table 1). Consequently, the findings of our analyses may not necessarily be applicable to recreational athletes.

## Conclusions

This systematic review and meta-analysis shows that, across team sports, ACL injuries most often arise without a direct force application to the knee, with cutting, defensive pressing or tackling, decelerating, and landing being the dominant high-risk actions. Being tackled predominates in contact injuries. Injuries commonly occur during ball possession and offensive play, and frequently at high horizontal velocity; in football and netball, injuries occur more often early in the match. From these results, sports-specific recommendations for ACL injury prevention can be informed.

However, the interpretation is tempered by the heterogeneity in reporting (overlapping categories, limited joint distributions of mechanism by movement by velocity), scarce exposure denominators, variable study quality, and an under‑representation of female athletes. Future work should harmonize taxonomies, provide injury case-level supplemental tables enabling joint analyses, include exposure and time-of-match data, and report on sex-specific differences and recreational athletes. These improvements will help align prevention with the dominant mechanisms and their situational contexts, offering a translational route to reduce ACL injury burden across team sports.

## Supplementary Information

Below is the link to the electronic supplementary material.

Supplementary Material in the online version includes the extracted data and the quality of the eligible studies. (DOCX 19 kb)

## Declarations

### Availability of data and material

All extracted information can be found in the figures and Supplementary Materials.
