# Supplementary material for: Situations Leading to Anterior Cruciate Ligament Injury in Sports: A Systematic Review with Meta-analysis
Source: Sports Med. 2026 Apr 18;56(9):2139–60. doi: 10.1007/s40279-026-02436-1 (PMC13499757; doi:10.1007/s40279-026-02436-1)
Supplement: Supplementary file 1 [file 40279_2026_2436_MOESM1_ESM.docx]

**Pooled injury proportions**

***Non-contact and indirect contact injuries***

After excluding 12 of the 39 studies that did not consider contact injuries from the analysis, the pooled proportion of *non-contact and indirect contact* ACL injuries was 80.5% (*Tab. 3*).

Regarding *movement patterns* (*Tab. 4*), the highest proportion was found for pressing/ tackling situations (41.8%), followed by situations involving ball action (34.8%), and cutting (38%).

The moderator analysis revealed an association between the type of sports and the proportions of landing (double and single-leg), pressing/ tackling, and movement with ball actions involved (p<.05; *Tab. 4*).

For landing, more injuries were reported in netball (81.1%; n = 1) than in basketball (29.6%; 95% CI: 19.7-40.6%; n = 7), handball (21.1%; n = 1), Australian and American football and Rugby (summed as contact-oriented-football, COF) (14.8%; 95% CI: 6.7-25.4%; n = 5), or football (12.3%; 95% CI: 8.4-16.8%; n = 10). For pressing/ tackling movements, injuries were more frequent in football (42.5%; 95% CI: 37.6-47.4%; n = 10) than in COF (20.5%; n = 1). For movements with ball action involved, more injuries were reported in basketball (52.7%; 95% CI: 36.4-70.7%; n =4) and in netball (52.7%; n =2), than in football (18.2%; 95% CI: 11.5-25.1%; n =8) or COF (8.7%; n=1).

In terms of the *time point of injury* (*Tab. 7*), overall traumas were most common in the last quarter (32.8%). The moderator analysis revealed that traumas in the first quarter of the game had higher injury proportions for football (45.5%; n = 2) than in netball (37.8%, n = 1), COF (24.7%; n = 2), or basketball (16.3%; n = 2).

Injuries at high horizontal *movement speed* (*Tab. 6*) were more frequent (53.0%) than with low (41.6%) or zero horizontal speed (5.1%). For vertical speed, most injuries were reported at no speed (56.4%), followed by low (25.9%) and high (17.1%) speeds. According to moderator analysis, the type of sport affected pooled injury proportions for vertical movements at low speed (p < .05; *Tab. 6*). Injuries were reported more frequently for basketball (58.5%; n=1), followed by football (23.0%; 95% CI: 12.5-35.7%; n =4), and COF (8.7%; n=1).

For different *game situations* (*Tab. 7*), injuries were more frequent in offensive play (51.7%) than during situations with ball possession (47.0%) or during defensive situations (38.1%). According to the moderator analysis, during defensive situations, pooled non-contact and indirect contact injury proportions were moderated by the type of sport (p<.05; *Tab. 7*). Football (62.5%; 95% CI: 55.1-69.6%; n =8) showed higher injury proportions than COF (33.0%; 95% CI: 9.1-63.1%; n =3), netball (22.0%; n=2), and basketball (15.0%; 95% CI: 6.2-26.8%; n =4).

***Direct and indirect contact injuries***

The pooled summary proportion for direct and indirect contact injuries combined was 56.7% (*Tab. 3*).

Regarding *movement patterns*, the proportions were highest for situations involving being tackled (37.2%), ball actions (23.1%), and combined double- and single-leg landings (22.8%). The moderator analysis revealed no significant effect of the type of sport on the pooled proportions of direct and indirect injuries (*Tab. 4)*.

In terms of horizontal *movement speed* (*Tab. 6),* higher proportions were found for high-velocity actions (28.3%), while low-speed (7.1%) and zero-speed actions (6.3%) were scarce. The moderator analysis was either not possible (small number of studies) or revealed no significant effect.

For *game situation* variables (*Tab. 7*), proportions were higher for offensive play (24.9%) than for defensive play (20.5%). Injuries were more frequent during the first quarter (26.6%). Moderator analysis revealed a significant effect of the type of sports and the injury proportions during defensive situations. (p<.05; *Tab. 7*). For all *game situation* variables, the moderator analysis was either not possible or did not show a significant effect.
